# Functional characterization of canine wild type glucocorticoid receptor and an insertional mutation in a dog

**DOI:** 10.1186/s12917-019-2129-9

**Published:** 2019-10-24

**Authors:** Kosei Yamanaka, Masaru Okuda, Takuya Mizuno

**Affiliations:** 1 Laboratory of Molecular Diagnostics and Therapeutics, Joint Faculty of Veterinary Medicine, Yamaguchi University, 1677-1 Yoshida, Yamaguchi-shi, Yamaguchi, 753-8515 Japan; 2 Laboratory of Veterinary Internal Medicine, Joint Faculty of Veterinary Medicine, Yamaguchi University, 1677-1 Yoshida, Yamaguchi-shi, Yamaguchi, 753-8515 Japan

**Keywords:** Canine, Iatrogenic Cushing syndrome, Glucocorticoid receptor, Reporter gene assay

## Abstract

**Background:**

Glucocorticoids, among the most widely utilized drugs in veterinary medicine, are employed to treat a wide variety of diseases; however, their use often induces adverse events in dogs. The efficacy of glucocorticoids usually depends on dosage, although differences in sensitivity to glucocorticoids in individual animals have been reported. Glucocorticoids bind to the cytoplasmic glucocorticoid receptor (GR), which is expressed in almost all cells. These receptors are key factors in determining individual sensitivity to glucocorticoids. This study examined individual differences in glucocorticoid sensitivity in dogs, focusing on reactivity of the GR to prednisolone.

**Results:**

We first molecularly cloned the *GR* gene from a healthy dog. We discovered a mutant *GR* in a dog suspected to have iatrogenic Cushing syndrome. The mutant *GR* had extra nucleotides between exons 6 and 7, resulting in a truncated form of GR that was 98 amino acids shorter than the wild-type dog GR. The truncated GR exhibited very low reactivity to prednisolone, irrespective of concentration.

**Conclusions:**

We have identified the truncated form of canine GR in a dog with iatrogenic Cushing syndrome. This truncated form showed the very less sensitivity to glucocorticoid *in vitro*, unfortunately, we could not elucidate its clinical significance. However, our data is a first report about the function of canine GR, and will facilitate the analysis of canine glucocorticoid sensitivity.

## Background

Glucocorticoids are used to treat numerous diseases, inflammatory conditions, transplant rejection, and various autoimmune disorders in both human and veterinary medicine. The primary expected effects of glucocorticoids are anti-inflammatory and immunosuppressive in nature and depend on the dose of medication. Generally, whereas lower doses of glucocorticoids induce only anti-inflammatory effects, higher doses are associated with additional immunosuppressive effects. However, even in the same species, individual differences in response to glucocorticoids can occur [1–3].

The functions of glucocorticoids are initiated upon binding to the cytoplasmic glucocorticoid receptor (GR), which is expressed in most types of cells. Because GRs are nuclear receptors, glucocorticoid/GR complexes are translocated to the nucleus, where they are involved in regulating the transcription of many genes. GRs have several important functional domains: an N-terminal regulatory domain (NTD), a DNA-binding domain (DBD), and a ligand-binding domain (LBD). Cloning and sequencing of guinea pig GR revealed 24 amino acid differences in the LBD compared to the human GR. Lower affinity of the guinea pig GR for dexamethasone in comparison with mouse and human GR was attributed to 4 of these 24 different amino acid residues [4]. In addition, guinea pigs have higher levels of circulating cortisol than mice and humans. Individual differences in sensitivity to glucocorticoids due to single nucleotide polymorphisms or GR structural abnormalities have been frequently reported in humans [1–3, 5].

Compared with humans, domesticated dogs and cats are relatively resistant to glucocorticoids [6]. However, the mechanism underlying this species-specific difference remains unclear, and its elucidation has awaited molecular characterization of the dog GR. In this study, we molecularly cloned the canine *GR* from three healthy dogs and a dog undergoing veterinary treatment for suspected iatrogenic Cushing syndrome. We determined the sensitivity of each GR to prednisolone using a reporter gene assay. In addition, we identified structural defects in the GR of the dog undergoing veterinary treatment and determined the GR sensitivity in this dog.

## Results

### Clinical description of a dog suspected of having iatrogenic Cushing syndrome

**Fig. 1:**
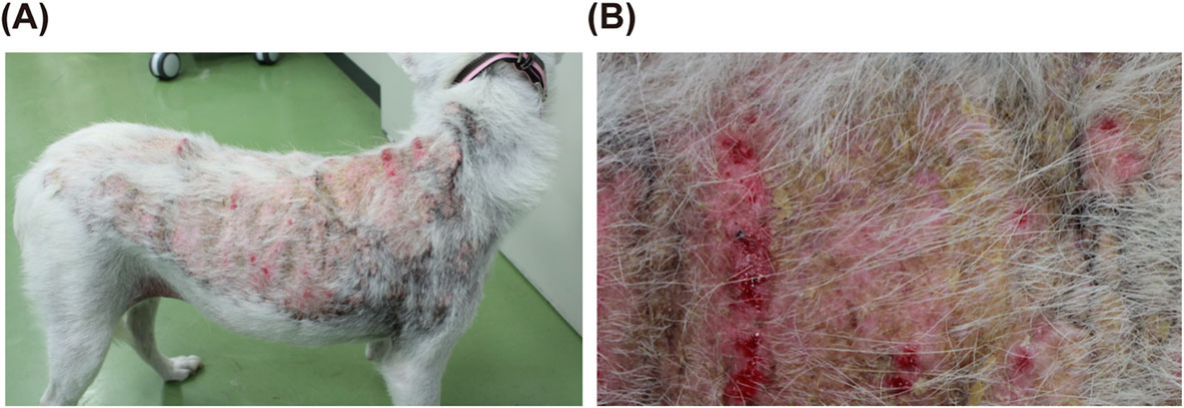
Bilateral hair loss on truncal regions and calcinosis in a dog with iatrogenic Cushing syndrome. **a** Alopecia and calcinosis cutis on the dorsal region of this dog. **b** Close-up image of lesions exhibiting calcinosis cutis in (**a**)

A 6-year-old spayed, mixed-breed dog was referred to the Yamaguchi University Animal Medical Center for detailed examination of skin disease. The dog was tentatively diagnosed with pemphigus foliaceus at a private hospital 9 months previously. The dog had been treated with prednisolone (0.7 mg/kg/day orally) for approximately 3 months. After the dog was started on the medication, polyuria, polydipsia, and abdominal distension were observed. The pemphigus foliaceus was neither ameliorated nor aggravated. After prednisolone withdrawal, calcinosis cutis was observed on the dorsal skin. Two months later, on the day of consultation at our hospital, the adverse reaction due to glucocorticoid therapy had already disappeared, except for extremely severe calcinosis cutis over the entire dorsal skin (Fig. 1a and b). Table 1 shows the results of a blood examination on this day.

**Table 1:** Hematological and biochemical findings in the dog on day 1

Red blood cells	853 × 10^4^ /μl	BUN	10.6 mg/dl
Hemoglobin	19.5 g/dl	Creatinine	0.6 mg/dl
Hematocrit	53%	AST	24 IU/l
Mean corpuscular volume	64.0 fl	ALT	52 IU/l
Mean corpuscular hemoglobin	22.9 pg	ALP	511 IU/l
Mean corpuscular hemoglobin concentration	35.7 g/dl	GGT	10 IU/l
		Albumin	3.0 g/dl
White blood cells	7329 /μl	Total Cholesterol	197 mg/dl
Band neutrophils	73 /μl	Glucose	99 mg/dl
Segmented neutrophils	5864 /μl	Na	148 mEq/l
Lymphocytes	586 /μl	N	3.8 mEq/l
Monocytes	220 /μl	Cl	114 mEq/l
Eosinophil	586 /μl	Ca	9.5 mg/dl
		IP	4.4 mg/dl
Platelet	44.0 × 10^4^ /μl	CRP	0.3 mg/dl
Total protein	7.0 g/dl		

### Nucleotide and deduced amino acid sequences of canine *GR * cDNA

**Fig. 2:**
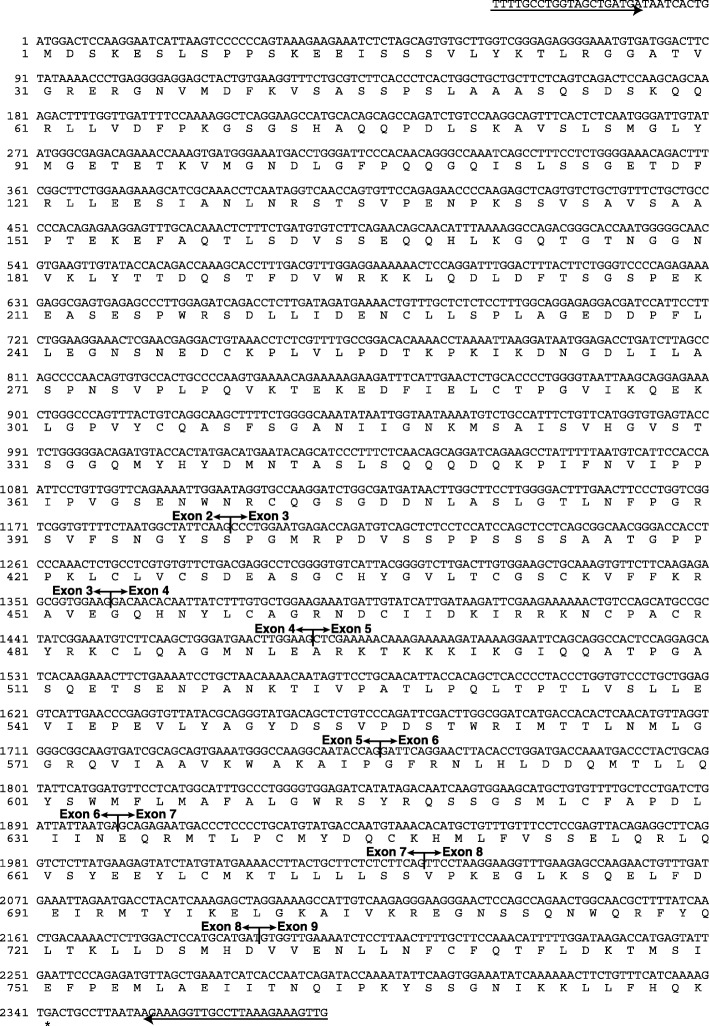
Nucleotide sequence and predicted amino acid sequence of dog *GR* cDNA. The nucleotide sequence of dog *GR* is shown, with the predicted amino acid sequence shown below the nucleotide sequence. Numbers to the left refer to the nucleotide position in the dog *GR* cDNA or amino acid position in the dog GR. Arrows indicate the primers used for cDNA cloning. The asterisk indicates the stop codon. The nucleotide sequence data reported in this paper were submitted to the DNA Data Bank of Japan (DDBL)/European Molecular Biology Laboratory (EMBL)/GenBank (Accession No. AB874470). Vertical lines indicate exon-exon boundaries

Before analyzing the nucleotide sequence of the dog’s *GR*, the nucleotide sequence of the canine *GR* was molecularly cloned and analyzed, as it had not been reported previously. PCR amplification of the dog *GR* cDNA (derived from one liver and two PBMCs all from healthy beagles) using the primers YTM673 and YTM674 provided a DNA fragment with an expected size of approximately 2500 bps. Nucleotide sequencing of the full-length dog *GR* revealed a cDNA clone covering 2522 bps and containing an open reading frame of 2343 bps encoding a protein of 780 amino acids (Fig. 2). The nucleotide sequence of the dog *GR* was 91, 87, and 89% identical to that of human *GRα* (GenBank Accession No. P04150), mouse (*Mus musculus*) *GRα* (GenBank Accession No. P06537), and guinea pig (*Cavia porcellus*) *GRα* (GenBank Accession No. P49115), respectively.

**Fig. 3:**
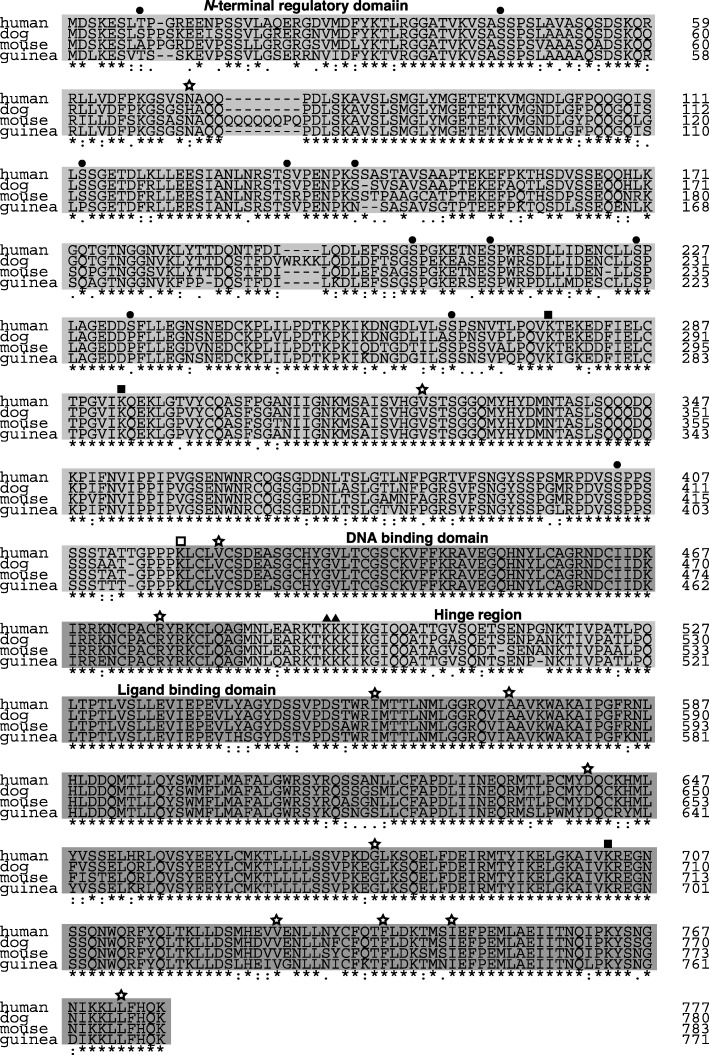
Comparison of the predicted amino acid sequences of GRs from different species. The amino acid sequence of the dog GR was aligned with that of human, mouse, and guinea pig GRs using Clustal W software. The “*” below columns in the amino acid sequences indicate that the residues in that column are identical in all sequences in the alignment. The “:” and “.” symbols indicate that semiconserved substitutions were observed. Numbers to the right refer to amino acid positions. GR amino acid sequences were obtained from the NCBI database (NP_000167.1 for human, P06537.1 for mouse, and NP_01166458.1 for guinea pig). The GR is composed of four domains, which are shown in shaded boxes: N-terminal regulatory domain, DNA-binding domain, hinge region, and ligand-binding domain. Each domain was defined according to the domain composition of the human GR. Closed circles, closed boxes, open box, and closed triangles indicate phosphorylation sites, SUMOylation sites, ubiquitination site, and acetylation sites, respectively, which were identified in the human GR. Stars indicate known mutation sites in the human GR associated with natural resistance

Alignment of the predicted amino acid sequence of the dog *GR* cDNA clones with those of the human, mouse, and guinea pig cDNAs is shown in Fig. 3. The deduced amino acid sequence of the dog GR cDNA exhibited 92, 89, and 88% homology with that of the human, mouse, and guinea pig GRα polypeptides, respectively. Consistent with the high homology of the predicted dog GRα amino acid sequence to those of other species, the dog GRα appeared to be composed of an N-terminal regulatory domain (NTD), a DNA-binding domain (DBD), a hinge region (HR), and a ligand-binding domain (LBD), similar to the human, mouse, and guinea pig receptors. These results suggest that the function of the dog GRα is similar to that of other species. Of the functional domains, the DBD was the most conserved between species, and no amino acid differences were found between the human, dog, and mouse GRαs. The LBD also exhibited high homology between species, whereas the NTD exhibited less homology.

Phosphorylation of the human GRα at many sites (T8, S45, S113, S134, S141, S203, S211, S226, S234, S267, and S404) has been reported, and this is considered to play an important role in GRα functions, such as transactivation and nuclear export [7–9]. Compared with the human receptor, most of the phosphorylation sites were conserved in dog and mouse GRα, except those at residues 8 and 234. The threonine at residue 8 was less conserved between species. The serine at residue 234 of the human GRα was changed to a proline in the mouse, dog, and guinea pig receptors.

In addition to phosphorylation, GRα undergoes other types of posttranslational modifications, such as acetylation, SUMOylation, and ubiquitination (Fig. 3) [10–12]. All reported modification sites in the human GRα were completely conserved in the mouse, dog, and guinea pig receptors.

A number of inactivating mutations (N72D, V321A, V423A, R477H, I559D, V571A, D641V, G679S, V729I, F737S, I747M, and L773P) in the human GRα gene are known to cause familial GC resistance [1–3] (Fig. 3). Compared with the inactivating mutations reported in the human GRα, most of sites were well conserved between the mouse, dog, and guinea pig receptor genes. However, the dog GR has a histidine at residue 72 rather than an asparagine.

### Nucleotide and deduced amino acid sequences of the GR cDNA of the treated dog

**Fig. 4:**
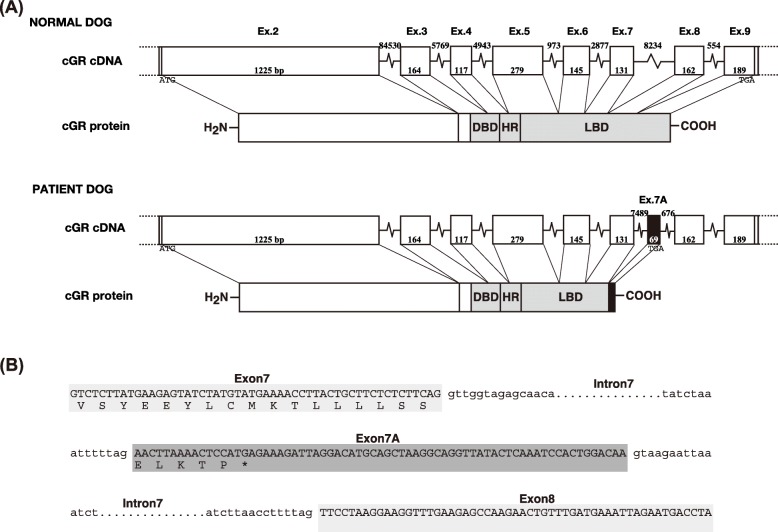
Structure of the GR gene in the dog with suspected iatrogenic Cushing syndrome. **a** Each box indicates an individual exon. Exon lengths are show in each box and intron lengths are indicated between each box. The extra exon present only in this dog is depicted in black. **b** Partial nucleotide sequence and predicted amino acid sequence of the cDNA of the dog treated in this case. The nucleotide sequence of the *GR* gene is shown, with the predicted amino acid sequence shown below the nucleotide sequence. The asterisk indicates the stop codon

PCR amplification of the cDNA of the dog treated at our hospital using the primers YTM673 and YTM674 generated a DNA fragment with a slightly larger size than an expected size. The nucleotide amino acid sequence of this *GR* clone was aligned with that of dog wild type *GR* (Fig. 2), which revealed that the *GR* of the dog undergoing treatment had an extra 69 nucleotides between nucleotides 2032 and 2033 compared with dog wild type *GR*. These extra 69 nucleotides matched a part of the nucleotide sequence of dog genomic DNA corresponding to intron 6 (Fig. 4a). Insertion of these extra 69 nucleotides between exons 6 and 7 introduced a frameshift and a premature termination codon (TGA) 15 bp downstream of the insertion. This insertion is thus predicted to result in a truncated protein of 682 amino acids, compared to the normal (wild type) 780 amino acids (Fig. 4b).

### Overexpression of dog GR in COS-7 cells

**Fig. 5:**
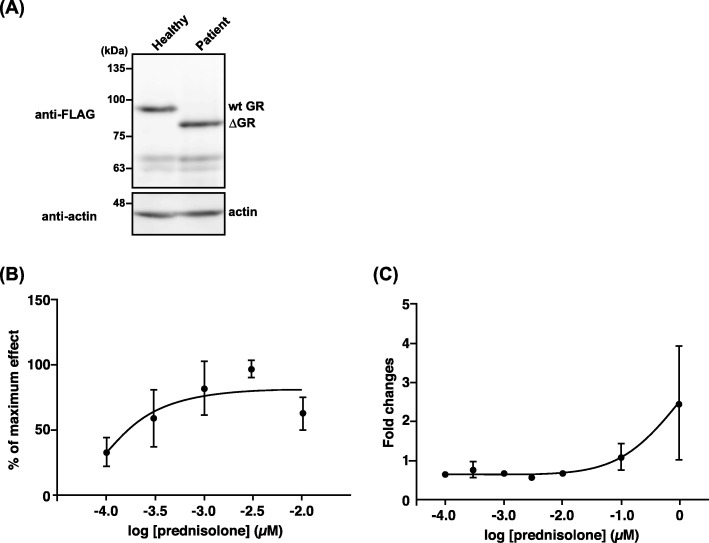
Expression and sensitivity of cGRΔLBD to prednisolone. **a** COS-7 cells were transiently transfected with pCR3-cGR#3 or a cGRΔLBD-expressing vector. Forty-eight hours after transfection, whole cell lysates were prepared and analyzed by western blotting using an anti-FLAG antibody (M2). The results of 1 of 2 comparable experiments are shown. **b**, **c** Lower responsiveness of cGRΔLBD to prednisolone. COS-7 cells were transiently transfected with pGL4.36, pRL-SV40, and either a cGR- or cGRΔLBD-expressing vector and then treated with different doses of prednisolone (10^− 4^ to 1 μM) for 24 h. Transactivation of cGR (**b**) or cGRΔLBD (**c**) was evaluated using a luciferase assay. Values were calculated as % of maximal effect (**b**) or fold-increase in reactivity to basal treatment (**c**) and represent the means ±SE of more than three independent experiments

To confirm the molecular size of the treated dog’s GR (cGR∆LBD) cloned in this study, FLAG-tagged cGR∆LBD or wild type GR were transfected into COS-7 cells, and the expression of the GR was examined by western blotting using an anti-FLAG antibody (Fig. 5a). A single band of the expected molecular weight and several bands of lower molecular weight were observed in COS-7 cells transfected with FLAG-tagged dog GR in COS-7 cells, whereas a band showing a lower molecular weight than wild type GR was confirmed in COS-7 cells transfected with the FLAG-tagged cGR∆LBD.

### Sensitivity of the treated dog’s GR to prednisolone

To compare the sensitivity to glucocorticoids of the treated dog GR and wild type GR, the luciferase activity of the glucocorticoid response element (GRE) of mouse mammary tumor virus (MMTV) was determined in COS-7 cells transfected with either *GRα* expression vector, followed by treatment with various concentrations of prednisolone (Fig. 5b and c). The concentration of prednisolone resulting in the maximum luciferase activity was 2.0 × 10^− 3^ μM for the normal, healthy dog (Fig. 5b). The calculated EC_50_ of dog GR–induced luciferase activity was 0.2 × 10^− 4^ μM.

To determine the effect of deletion of the LBD in cGR∆LBD on glucocorticoid sensitivity, the luciferase activity of the GRE was determined in COS-7 cells transfected with cGR∆LBD and treated with various concentrations of prednisolone. The luciferase activity of cGR∆LBD was very low, irrespective of the concentration of glucocorticoid, and unaltered by any increase in the prednisolone concentration (10^− 4^ to 10^− 2^ μM). However, the activity increased slightly at very high prednisolone concentrations (10^− 1^ to 1 μM) (Fig. 5c).

## Discussion

In this study, we first isolated the dog *GR* gene from a healthy normal dog and compared its glucocorticoid sensitivities to that of the *GR* of a dog being treated at our hospital. The dog was being treated for suspected iatrogenic Cushing syndrome, and its GR was characterized by a deletion, which was designated cGR∆LBD. We were not able to obtain a complete history regarding the clinical symptoms and medications for this dog, but treatment with prednisolone had not been for an extended period, and the dosage did not seem too high based on the limited information available to us. We then considered that this dog could be very sensitive to prednisolone and hypothesized that the abnormal GR was more sensitive to glucocorticoid than normal dog GR. cGR∆LBD had a shorter LBD than the normal dog LBD due to alternative splicing generated by insertion of an extra exon. Furthermore, cGR∆LBD exhibited poor sensitivity to prednisolone, in complete contradiction to our hypothesis. On the one hand, this minimal sensitivity of cGR∆LBD to glucocorticoid was consistent with the results of a previous study showing that an artificial LBD-deletion mutant human GR had no sensitivity to glucocorticoid [13]. The LBD plays a role in the binding of glucocorticoid to the GR, and its sensitivity varies. For example, the guinea pig GR has lower affinity for dexamethasone than the mouse and human GRs [14] due to 4 important differences in the amino acid sequence between residues 544–551 of the LBD. Asparagine 619, leucine 620, tyrosine 735, and asparagine 766 in the LBD of hGR are conserved only in the human and mouse GR, but in the dog GR, these residues are substituted with serine, methionine, phenylalanine, and serine, respectively. These differences could contribute the increased sensitivity of dog GR to prednisolone as compared with the human and mouse GRs.

In humans, no or little sensitivity to glucocorticoid is known as cortisol resistance and typically results from a mutation in the LBD [1]. Among these mutations, I559N and I747M are known as dominant negative mutations [15], which lead to clinical syndromes such as cortisol resistance. Patients with these mutations present with increased cortisol levels compared with the normal population. The negative glucocorticoid feedback controlling secretion of both corticotrophin-releasing hormone (CRH) and adrenocorticotrophin (ACTH) is suppressed as a consequence of diminished glucocorticoid sensitivity. Secretion of both CRH and ACTH increases, resulting in higher serum cortisol levels, which appear to compensate adequately for the reduced sensitivity. As the increased serum cortisol levels are physiologically needed, patients do not show symptoms of cortisol excess [16]. The diagnostic criteria for generalized glucocorticoid resistance include no clinical signs of Cushing syndrome, increased plasma total and free cortisol (normal plasma cortisol-binding globulin concentration), 24-h urinary free cortisol or 17-hydroxysteroid excretion, increased rate of cortisol production, and resistance to dexamethasone [17]. As we did not perform all of these examinations, we cannot completely exclude the possibility that this dog had cortisol resistance. However, it is unlikely that this dog had generalized cortisol resistance because it did not exhibit increased plasma cortisol (baseline cortisol of 1.5 μg/dL and 6.18 μg/dL at 3 weeks and 17 months after the first visit to our hospital, respectively) but did exhibit increased alkaline phosphatase after treatment with prednisolone at the private veterinary hospital.

The discrepancy between this dog’s clinical response to glucocorticoid and harboring a glucocorticoid-unresponsive form of GR could be explained as follows. Whereas human patients exhibiting generalized glucocorticoid resistance have genomic mutations in the LBD of the GR, the treated dog of this case harbored an alternative splicing variant *GR*. This could occur only in peripheral blood mononuclear cells (PBMCs) from this dog; if other tissues had GRs with a normal splicing pattern, generalized glucocorticoid resistance should not be observed. Unfortunately, we were not able to obtain other tissues from this dog and thus could not determine the expression pattern of *GR*s in other tissues. Moreover, we were not able to obtain genomic DNA from this dog, which could have provided information as to whether this mutant form of *GR* derived from germ-line mutations that could induce the abnormal splicing pattern. In this study, we also cloned the *GR* from the PBMCs from two normal healthy dogs, as well as the thymus of a normal, healthy dog. On the other hands, this dog had only mutant form of *GR* in PBMC. This suggested that the mutant form of GR, cGR∆LBD, is characteristics of *GR* in PBMC from this dog.

In this study, we conducted a molecular characterization of the dog GR. We also identified a natural mutant GR in a dog being treated for suspected iatrogenic Cushing syndrome and compared its function with that of the GR of a normal, healthy dog. The precise mechanism controlling the sensitivity of the GR in dogs remains to be elucidated, but the results of this study could facilitate further studies of dog GR sensitivity and/or resistance.

## Conclusions

In this study, we molecular cloned canine *GR* for the first time. We also identified a mutated *GR* from the dog with iatrogenic cushing’s syndrome. This mutated *GR* had very low sensitivity to glucocorticoid treatment as shown by GR-responsive reporter gene assay. Although the significance of this mutated *GR* in iatrogenic cushing’s syndrome is still unknown, but this is the first report about abrogated canine GR from patient dog.

## Methods

### Cell culture

COS-7 (African Green monkey kidney) cells were grown in R10 complete medium (RPMI 1640; Nacalai Tesque, Kyoto, Japan) supplemented with 10% fetal calf serum, 55 μM 2-mercaptoethanol, 100 units/ml penicillin, and 100 μg/ml streptomycin. Cells were incubated at 37 °C in a humidified atmosphere containing 5.0% CO_2_.

### Molecular cloning of canine *GR*s

A whole blood was obtained from a dog undergoing veterinary treatment under the dog’s owner consent, and was used to clone the full-length dog GR. One microgram of total RNA was isolated from each specimen, treated with Turbo DNA-free (Ambion Life Technologies, CA), and then transcribed into cDNA using Superscript III (Invitrogen Life Technologies, CA), according to the manufacturer’s instructions. Oligo dT primers were used to prime the first-strand synthesis for each reaction. For a control dog sample, the frozen stocked cDNA of canine thymus and PBMCs in previous study {Mizuno:2009tw} was used.

The primers to amplify dog *GR* cDNA, YTM673 (5′-TTTTGCCTGGTAGCTGATGA-3′) and YTM674 (5′-CAACTTTCTTTAAGGCAACCTTTC-3′), were designed based on predicted sequences of the *GR* as determined from the dog genomic database (GenBank Accession No. NW_876266.1). Using these primers, the dog *GR* gene was amplified from cDNA using a KOD plus kit (Toyobo, Osaka, Japan) according to the manufacturer’s instructions. Pre-denaturation at 94 °C for 2 min was followed by 35 cycles of PCR amplification consisting of denaturation at 94 °C for 10 s, annealing at 60 °C for 30 s, extension at 68 °C for 3 min, and then a final extension at 68 °C for 10 min using a PCR System 9700 (Perkin-Elmer, Waltham, MA). Each gel-purified PCR product was inserted into *Sma*I restriction sites of the pBluescript SK (−) vector (designated pBS-GRα full#1). The constructed insert-containing vector was sequenced using M13 (− 20) forward primer and M13 reverse primer with a BigDye Termination v3.1 Cycle Sequencing kit (Perkin-Elmer) and analyzed using a PRISM3100-Avant sequencer (Applied Biosystems, Foster City, CA) at the Yamaguchi University Center for Gene Research. The resulting sequencing data were compared to the previously reported *GR* using ApE (A plasmid Editor) and Clustal W software.

### Construction of expression vectors

To obtain the vector encoding the dog *GR*, two FLAG-tag sequences were added at the N-terminus of the *GR*. PCR was carried out with each primer pair, YTM1033 (5′-AGGAATTCGACTCCAAGGAATCATA-3′) and YTM1034 (5′-CCAGAGGAAAGGCTGATTTG-3′), using pBS-GRα full#1 as a template. The PCR product was purified and digested with *Bgl*I and *Eco*RI. To obtain the second half of the cGR, pBS-GRα full#1 was digested with *Bgl*I and *Xba*I. The amplified product carrying the FLAG tag and the corresponding second half digested with restriction enzymes were ligated into the *Eco*RI and *Xba*I sites of the pCR2xFL vector. The nucleotide sequence of the clone was confirmed by sequencing, as described above.

### Western blot analysis

For transfection, cells were plated in a 6-well plate at 3.0 × 10^5^ cells in 2 ml of R10 medium 24 h prior to transfection. Cells were transfected using TransIT-LT1 (Mirus Bio Corporation, WI) transfection reagent according to the manufacturer’s instructions. In brief, 1 μl of TransIT-LT1 and 1 μg of one of the GR expression vectors were mixed in 100 μl of un-supplemented RPMI 1640 medium. After incubation for 15 min at room temperature (RT), the mixture was added to the cells. At 48 h after transfection, the cells were washed with D-PBS(−) and stored at − 80 °C until use.

For western blot analysis, cells were pelleted and then lysed with 1% NP40 lysis buffer (150 mM NaCl, 10 mM Tris-HCl [pH 7.5], 1 mM EDTA, 1 mM PMSF, and complete Mini EDTA-free protease inhibitor mixture [Roche Diagnostics K.K., Tokyo, Japan]), centrifuged at 15,000 rpm for 15 min at 4 °C, and then the supernatant was collected. Extracted proteins were separated by 8% SDS-PAGE and then transferred onto a PVDF membrane (Hybond-P GE Healthcare, Little Chalfont, U.K.). The membrane was blocked with 5% nonfat dry milk in 1× TBS-T (1× TBS, 0.05% Tween-20) for 1 h at RT and then incubated with anti-FLAG antibody (clone M2, 1:1000; Sigma-Aldrich, St. Louis, MO) overnight at 4 °C. The membrane was then washed three times with TBS-T for 10 min and incubated with horseradish peroxidase–conjugated goat anti-mouse IgG polyclonal secondary antibody (Zymed, South San Francisco, CA) (1:4000 dilution) for 1 h at RT. The membrane was washed three times with TBS-T for 10 min each and visualized by immersion in Western Lightning Chemiluminescence reagent (Perkin–Elmer). Immunoreactive bands were quantified using a Las 3000 mini luminescent image analyzer (Fujifilm, Tokyo, Japan), and the data were analyzed using Science Lab 2005 (Fujifilm). Immunoblots were then stripped and re-probed with anti-actin antibody (clone C-11, 1:1000; Santa Cruz Biotechnology, Inc).

### Luciferase assay

At 24 h prior to transfection, cells were plated at 2.0 × 10^5^ cells in 1 ml of R10 medium in a 12-well plate. Cells were transfected using TransIT-LT1 (Mirus Bio Corp.) transfection reagent. In brief, 3 μl of TransIT-LT1, 330 ng of pGL-4.36 (Promega, Madison, WI), 10 ng of pRL-SV40 (Promega), and 670 ng of one of the GR-expressing vectors in 100 μl of Opti-MEM serum-free medium (Gibco®, Invitrogen Life Technologies, CA) were mixed. Twenty-four hours after transfection, cells were detached and re-plated in a 96-well plate (2.0 × 10^4^ cells in 75 μl of R10 medium) in triplicate. Cells were incubated for an additional 24 h, and then serial dilutions of prednisolone were added to each well. After 24 h of incubation, the cells were lysed using a Dual-Glo™ Luciferase Assay System (Promega), and luciferase activity was assessed using a DTX800 Multimode Detector (Beckman Coulter®, Brea, CA) according to the manufacturer’s instructions. Firefly luciferase activity was normalized to renilla luciferase activity generated from the pRL-SV40 vector in the same cells. EC_50_ values were calculated based on concentration-response curves using GraphPad Prism6 software (GraphPad, Inc., La Jolla, CA).

## Data Availability

The datasets used and/or analyzed during the current study are available from the corresponding author on reasonable request.

## Abbreviations

ACTH: Adrenocorticotrophin
CRH: Corticotrophin-releasing hormone
DBD: DNA-binding domain
GR: Glucocorticoid receptor
GRE: Glucocorticoid response element
LBD: Ligand-binding domain
MMTV: Mouse mammary tumor virus
NTD: N-terminal regulatory domain
PBMCs: Peripheral blood mononuclear cells
SD: Standard deviation

## References

[1] Charmandari E, Kino T, Ichijo T, Chrousos GP (2008). Generalized glucocorticoid resistance: clinical aspects, molecular mechanisms, and implications of a rare genetic disorder. J Clin Endocrinol Metab Endocrine Soc.

[2] Baker AC, Chew VW, Green TL, Tung K, Lim D, Cho K (2013). Single nucleotide polymorphisms and type of steroid impact the functional response of the human glucocorticoid receptor. J Surg Res.

[3] Roberts ML, Kino T, Nicolaides NC, Hurt DE, Katsantoni E, Sertedaki A (2013). A novel point mutation in the DNA-binding domain (DBD) of the human glucocorticoid receptor causes primary generalized glucocorticoid resistance by disrupting the hydrophobic structure of its DBD. J Clin Endocrinol Metab.

[4] Keightley MC, Fuller PJ (1994). Unique sequences in the Guinea pig glucocorticoid receptor induce constitutive transactivation and decrease steroid sensitivity. Mol Endocrinol.

[5] Trebble P, Matthews L, Blaikley J, Wayte AWO, Black GCM, Wilton A (2010). Familial glucocorticoid resistance caused by a novel Frameshift glucocorticoid receptor mutation. J Clin Endocrinol Metab.

[6] Lowe AD, Campbell KL, Graves T (2008). Glucocorticoids in the cat. Vet Dermatol.

[7] Yang N, Ray DW, Matthews LC (2012). Current concepts in glucocorticoid resistance. Steroids..

[8] Krstic MD, Rogatsky I, Yamamoto KR, Garabedian MJ (1997). Mitogen-activated and cyclin-dependent protein kinases selectively and differentially modulate transcriptional enhancement by the glucocorticoid receptor. Mol Cell Biol.

[9] Dephoure N, Zhou C, Villén J, Beausoleil SA, Bakalarski CE, Elledge SJ (2008). A quantitative atlas of mitotic phosphorylation. Proc Natl Acad Sci U S A.

[10] Garside H, Waters C, Berry A, Rice L, Ardley HC, White A (2006). UbcH7 interacts with the glucocorticoid receptor and mediates receptor autoregulation. J Endocrinol.

[11] Beck IME, Vanden Berghe W, Vermeulen L, Yamamoto KR, Haegeman G, De Bosscher K (2009). Crosstalk in inflammation: the interplay of glucocorticoid receptor-based mechanisms and kinases and phosphatases. Endocr Rev.

[12] Ito K, Yamamura S, Essilfie-Quaye S, Cosio B, Ito M, Barnes PJ (2006). Histone deacetylase 2-mediated deacetylation of the glucocorticoid receptor enables NF-kappaB suppression. J Exp Med.

[13] Hollenberg SM, Giguere V, Evans RM (1989). Identification of two regions of the human glucocorticoid receptor hormone binding domain that block activation. Cancer Res.

[14] Keightley MC, Fuller PJ (1995). Cortisol resistance and the Guinea pig glucocorticoid receptor. Steroids..

[15] Kino T, Vottero A, Charmandari E, Chrousos GP (2002). Familial/sporadic glucocorticoid resistance syndrome and hypertension. Ann N Y Acad Sci.

[16] Lamberts SWJ (2002). Glucocorticoid receptors and Cushing's disease. Mol Cell Endocrinol.

[17] Arai K, Chrousos GP (1994). Hormone-nuclear receptor interactions in health and disease. Glucocorticoid resistance. Baillieres Clin Endocrinol Metab.

